# Assessment of Knowledge Regarding Safety Profile, Use, and Boxed Warnings of Fluoroquinolones Among Healthcare Professionals in Saudi Arabia: A Potential Implication for Drug Regulatory Authorities

**DOI:** 10.3389/fmed.2022.816320

**Published:** 2022-04-29

**Authors:** Tauqeer Hussain Mallhi, Abdullah Salah Alanazi, Yusra Habib Khan, Nasser Hadal Alotaibi, Muhammad Salman, Abdulaziz Ibrahim Alzarea, Salah-Ud-Din Khan, Nabil K. Alruwaili, Alaa Salah Alenazi, Ahmed D. Alatawi, Zafar Iqbal, Muhammad Hammad Butt, Muhammad Shahid Iqbal

**Affiliations:** ^1^Department of Clinical Pharmacy, College of Pharmacy, Jouf University, Sakaka, Saudi Arabia; ^2^Health Sciences Research Unit, Jouf University, Sakaka, Saudi Arabia; ^3^Faculty of Pharmacy, The University of Lahore, Lahore, Pakistan; ^4^Department of Biochemistry, College of Medicine, Imam Mohammad Ibn Saud Islamic University, Riyadh, Saudi Arabia; ^5^Department of Pharmaceutics, College of Pharmacy, Jouf University, Sakaka, Saudi Arabia; ^6^Riyadh Second Health Cluster, Ministry of Health, Rayyad, Saudi Arabia; ^7^Department of Pharmaceutical Services, Armed Forces Hospital, King Abdulaziz Airbase, Dhahran, Saudi Arabia; ^8^Department of Clinical Pharmacy, Faculty of Pharmacy, University of Central Punjab, Lahore, Pakistan; ^9^Department of Clinical Pharmacy, College of Pharmacy, Prince Sattam bin Abdulaziz University, Al-Kharj, Saudi Arabia

**Keywords:** anti-infective agents, antibiotics, fluoroquinolones, boxed warnings, United States Food and Drug Administration (FDA), safety, adverse effects, antibiotic stewardship

## Abstract

**Background:**

Despite a series of “boxed warnings” (BWs) issued by the US Food and Drug Administration (FDA), fluoroquinolones (FQs) are among the most prescribed antibiotics across the world. Moreover, few studies demonstrated that BW of FQs had less or no impact on prescribing patterns among healthcare professionals (HCPs), which might be attributed to the lack of knowledge toward such warnings. Since FQs contribute to a major proportion of antimicrobial prescriptions in the Kingdom of Saudi Arabia (KSA), this study aimed to ascertain the extent of knowledge toward safety profile, use, and BW of FQs among HCPs working in the KSA.

**Methods:**

This cross-sectional study (May–August 2021) was conducted among HCPs working in KSA through a validated questionnaire. The HCPs were requested to identify the indications, adverse effects (AEs), and BW of FQs. The knowledge score (out of 40) was estimated among participants, and its association with demographics was ascertained through the chi-square test, Student's *t*-test, or Mann-Whitney *U*-test and one-way ANOVA, or Kruskal-Wallis test, where appropriate.

**Results:**

Of the 573 participants (age: 36.1 ± 10.6 years, men: 59.7%), 262 (45.8%) were prescribers reporting frequent use of ciprofloxacin, levofloxacin, and ofloxacin. One-fourth (25.6%) of the prescribers did not recognize nalidixic acid as an agent from FQs class. About 60% of participants correctly identified the mechanism of action of FQs. The average knowledge score was 14.8 ± 6.4, where only 21.5% of respondents scored ≥50%. The average knowledge score for indications, AEs, and BW domains was 5.29 ± 3.05, 6.17 ± 4.05, and 2.3 ± 1.5, respectively. Only 75 (13.1%) participants recognized half of the BW, and 38.6% of participants identified at least one warning. The HCPs aged >40 years (*p* = 0.043), having non-Saudi's nationality (*p* < 0.001), working in Riyadh and Eastern regions (*p* < 0.001), having pharmacy and medicine disciplines (*p* < 0.001), practicing in public sectors (*p* = 0.004), and having more than 10 years of experience (*p* < 0.001) were significantly associated with high knowledge score.

**Conclusion:**

This study demonstrates the unsatisfactory knowledge toward safety profile, use, and BW of FQs among HCPs which may put patients at increased risks of AEs. The knowledge score differed among various socio-demographic groups. There is a dire need to initiate the antimicrobial-focused educational campaigns among HCPs regardless of their specialties and methods to improve education and disseminate FDA warnings in practice.

## Introduction

Antibiotics contribute to a substantial proportion of prescriptions for both inpatients and outpatients (1). Approximately, half of the patients admitted to the hospital receive antibiotics, particularly for pneumonia and urinary tract infection (UTI) (2). According to an estimate, about 20% of antibiotic encounters are associated with adverse effects (AEs) (3). Moreover, the lack of drug-related knowledge is found to be significantly correlated with medication errors (MEs). Fluoroquinolones (FQs) are widely used against various bacterial infections and are associated with severe AEs and antimicrobial resistance (2). The US Food and Drug Administration (FDA) has announced several “boxed warnings” (BWs) against FQs and limited their use according to the risks vs. benefits ratio. The first BW against FQs was issued in 2008 that included potentially permanent AEs of tendons, muscles, joints, nerves, and central nervous system (CNS). Subsequently, a series of warnings were announced, including worsening of myasthenia gravis (February 2011), irreversible peripheral neuropathy (August 2013), limiting the use of FQs for complicated infections (November 2015), risks of hypoglycemic coma, mental health AEs (July 2018), and aortic aneurysm (December 2018) (4, 5). It is pertinent to mention that AEs from FQs leading to emergency department visits occur at a rate of 9.2 per 10,000 prescriptions, even higher than macrolides and cephalosporins (6). Current antimicrobial guidelines also discourage the use of FQs as the first-line agents unless benefits overweigh the risks (7).

Despite the series of BW and a substantial number of evidence on toxicities, FQs are still prescribed irrationally in various healthcare settings (7). Although the declining trend of FQs prescriptions following the BW has been observed in a few studies from the United States (8, 9), still these warnings did not impact the prescribing pattern in various countries around the world (5, 7, 10–12). Since the violation of BW may pose serious health hazards to the patients, it has been observed that about 7 of 1,000 outpatients received prescriptions violating the BW, and a substantial number of these patients are at risk of developing serious AEs (13). The FQs are frequently used for various infections in the Kingdom of Saudi Arabia (KSA) (14), where ciprofloxacin is frequently prescribed among the outpatients (15–17). In addition, the widespread use of ciprofloxacin in KSA has been observed among all patients regardless of their age (18). The recent estimates from KSA showed that the FQs accounted for 5% of antibiotics prescribed in the emergency department, 22% of prescriptions for UTIs, 19% of antimicrobial prescriptions in ambulatory care, and 19% of antibiotic prescriptions in outpatient departments (1). The excessive or irrational use of FQs might be associated with the lack of awareness and knowledge among healthcare professionals (HCPs) regarding the safety profile, prescribing guidelines, and BW. Ascertaining the level of knowledge toward FQs among HCPs is of paramount importance to design and implement the targeted educational interventions and to preserve the utility of these drugs. In this context, this study was aimed to evaluate the awareness and extent of knowledge among HCPs (e.g., physicians, pharmacists, dentists, and nurses) toward safety profile, BW, and use of FQs. The findings of this study will underscore and strengthen the need for continued education among HCPs along with system-based interventions to reduce the inappropriate prescribing of these drugs.

## Methodology

### Ethics Statement

This study was approved by the Local Committee of Bioethics (LCBE) at Jouf University, KSA (Ref. 05-08-42). Informed consent was obtained from all participants. All the data were anonymized before analysis.

### Study Design and Location

This cross-sectional quantitative survey was conducted among HCPs working in the four provinces of KSA. The survey interviews were administered between May 2021 and August 2021. The four provinces included in this study were Riyadh, Eastern, Al-Jouf, and Northern border. The first two provinces are developed regions in the country, while the last two provinces are considered as least developed regions with limited healthcare facilities. Data collection from these four provinces was intended to ascertain the knowledge among HCPs across regions with maximum and limited health facilities.

### Study Population

**Figure 1:**
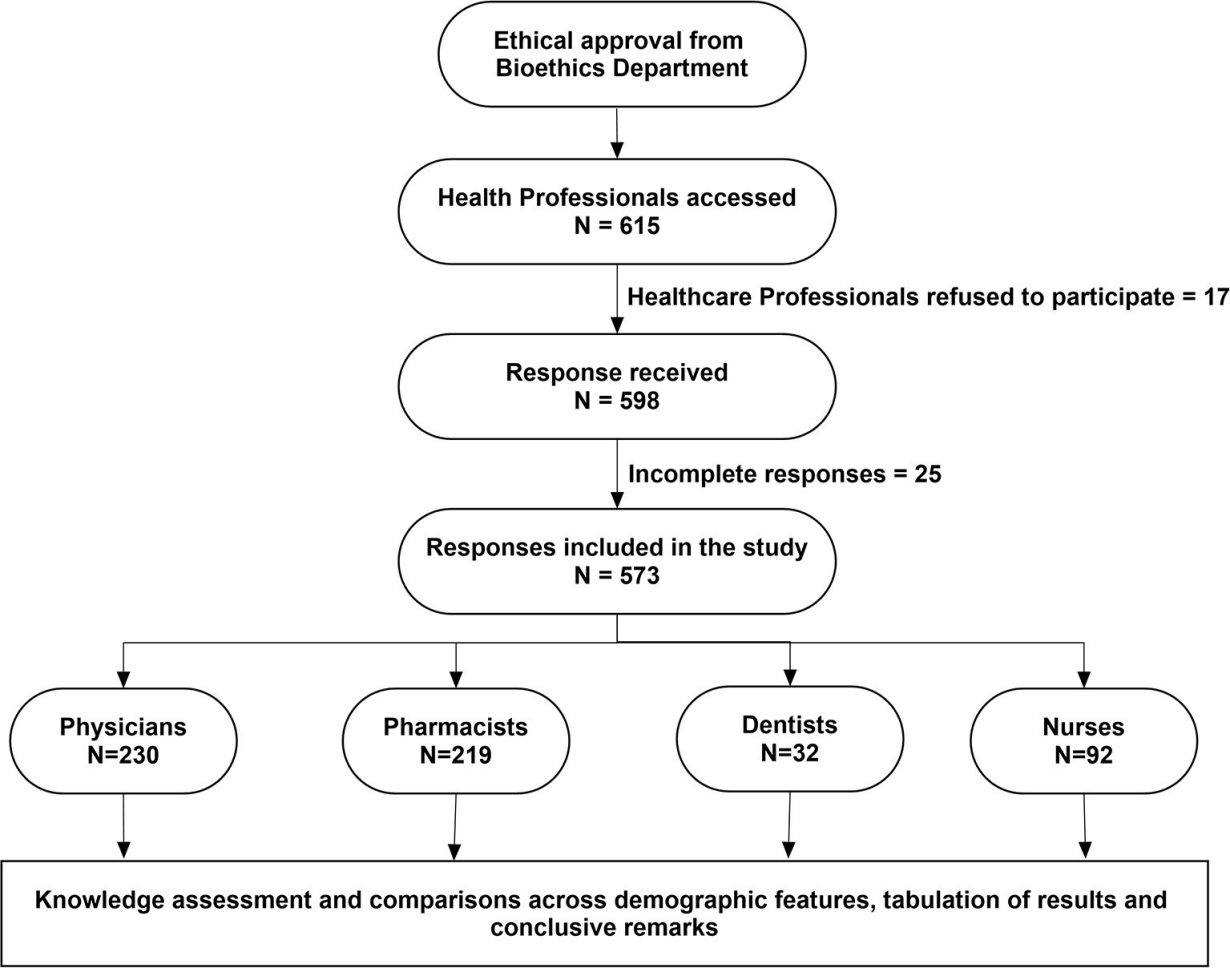
Study flow diagram.

The HCPs (e.g., physicians, pharmacists, nurses, and dentists) working in KSA were included in this study. The participants who were willing to participate and have active jobs with at least 1 year of working experience were included in this study. Only participants with an active license of practice from the health commission were included. Healthcare students, professionals having <1 year of experience, and those who refused to participate were excluded from the study. The study flow diagram for this study is presented in Figure 1.

### Validation and Reliability of Study Instrument

A 62-item questionnaire comprised of five sections was constructed under the opinions of experts from four health specialties (i.e., physicians, hospital/community pharmacists, dentists, and nurses). Following the face and content validity, the study instrument was administered in a small targeted sample of HCPs (*n* = 60) having equal distribution of all four specialties (15 participants from each specialty). The internal consistency of the study tool was assessed by alpha value which was found at 0.821, indicating the adequacy and reliability of the tool to evaluate the study objectives.

### Components of Study Instrument

All five sections in the study instrument had closed-ended questions. “Introduction” section had 9 items on demographics, including age, gender, qualification, working sector, experience, and nationality. “Methodology” section comprised names of 13 drugs from FQs class and only prescribers (e.g., physicians and dentists) were asked to respond their prescribing frequency of these drugs on a four-item scale (i.e., no use, frequent use, less frequent use, and new drug). Moreover, this section had one multiple-choice knowledge question on the mechanism of action of FQs, where the correct answer (DNA gyrase/topoisomerase IV inhibitor) was scored “1;” otherwise, scored “0.” “Results” section had a list of twelve indications of FQs. All the HCPs were asked to respond to these questions on a scale of “Yes,” “No,” and “Not Sure.” Since FQs are prescribed for all listed indications, the option “Yes” was scored “1”; otherwise, scored “zero.” Similarly, “Discussion” section had a list of eighteen AEs of FQs, and the HCPs were asked to respond each on a scale of “Yes,” “No,” and “Not Sure.” As these AEs are associated with the use of FQs, the participants who selected “Yes” were scored “1;” otherwise, scored “zero.” “Conclusion” section had nine items evaluating the participant's awareness and knowledge toward the BW of FQs. The respondents were requested to answer whether they know that the most recent BW against FQs was issued in 2018 by the FDA. The option “Yes” was scored “1;” otherwise, scored “zero.” In addition, “Conclusion” section had a list of eight BWs of FQs, and HCPs were asked to recognize each on a scale of “Yes,” “No,” and “Not Sure.” Since all these BWs are announced by the FDA from 2008 to 2018, the option “Yes” was scored “1;” otherwise, scored “zero.” Taken together, this study instrument had 40 items assessing the knowledge of HCPs toward the mechanism of action, indications, AEs, and BW of FQs, yielding a cumulative knowledge score of 40. The average knowledge score was also estimated for each item. Considering the fact that knowledge items in the study tool were in detail and had a high difficulty level, a 50% score was stratified as an “appropriate knowledge.”

### Data Collection

Using a convenient sampling technique, all the authors were asked to contact the HCPs from public or private health facilities, including hospitals and pharmacies, in four provinces of KSA. Informed consent was obtained from each participant before administering the survey. A brief overview was given to HCPs on the safety profile of FQs after collecting the questionnaire. All the questionnaires were checked for completeness and transferred to the Microsoft spreadsheet for cleaning purposes.

### Statistical Analysis

All the data were subjected to Statistical Package for the Social Sciences (SPSS version 22.0). The continuous variables were presented as mean with standard deviation or median with interquartile range (25–75%). The categorical data were described in frequencies (N) along with proportion (%). The mean knowledge score across various healthcare specialties (e.g., physicians, pharmacists, dentists, and nurses) was compared by one-way ANOVA or Kruskal-Wallis test, where appropriate. Moreover, the *post-hoc* analysis was also performed to ascertain the specific differences of knowledge score between these specialties. The Student's *t*-test or Mann-Whitney *U*-test was used to compare the knowledge score among demographics having two categories (bivariate), i.e., age, gender, working sector, and Saudi vs. Non-Saudi. In contrast, the knowledge score among demographics having more than two categories was compared through one-way ANOVA or Kruskal-Wallis test, where appropriate. In addition, we performed binary logistic regression to determine the factors or predictors (e.g., independent variables: age, gender, nationality, field of education, working location and sector, level of education, and working experience) associated with participants having knowledge scores ≥50% (dependent variable). The significant variables from the univariate analysis were considered for multivariate regression. These data were presented as odds ratio (OR) with 95% confidence interval (CI). A significance level of 0.05 was considered throughout the statistical analysis.

## Results

### Characteristics of the Study Participants

Out of the 615 HCPs who were approached, 573 HCPs were included in the final analysis, yielding a response rate of 93.1% (Figure 1). The mean age of the participants was 36.1 ± 10.6 years [median (interquartile range): 34 (17)] with male preponderance (59.7%). Majority of the respondents were Saudis (64.9%) followed by Egyptians (17.3%). The study population was physicians (40.1%), pharmacists (38.2%), dentists (5.6%), and nurses (16.1%). About 80% of respondents were working in the public sectors, including primary, secondary, and tertiary care hospitals, while 19.9% were from private hospitals and community pharmacies. The demographic features of the study participants are described in Table 1.

**Table 1:** Demographics of study participants (*n* = 573).

	**Frequency (*N*)**	**Percentage (%)**
**Age**		36.1 ± 10.6 (34, IQR: 45–28)
20–40 years	389	67.9
>40 years	184	32.1
**Gender**
Male	342	59.7
Female	231	40.3
**Nationality**
Saudis	372	64.9
Egyptians	99	17.3
Sudanese	25	4.4
Pakistanis	27	4.7
Indians	39	6.8
Syrians	11	1.9
**Saudis vs. non-Saudis**
Saudis	372	64.9
Non-Saudis (Expatriates)	201	35.1
**Province region**
Al-Jouf region	280	48.9
Riyadh region	107	18.7
Eastern region	112	19.5
Northern Border region	74	12.9
**Field of education**
Medicine (MBBS/MD)	230	40.1
Pharmacy	219	38.2
Dentistry	32	5.6
Nursing	92	16.1
**Level of education**
Graduation	348	60.7
Master	121	21.1
Doctorate	104	18.2
**Country of graduation**
Saudi Arabia	337	58.8
Egypt	97	16.9
India	39	6.8
Pakistan	25	4.4
Sudan	23	4.0
Syria	11	1.9
UK	18	3.1
USA	23	4.0
**Working sector**
Government sector	459	80.1
Private sector	114	19.9
**Working organization**
Tertiary care hospital	209	36.5
Secondary hospital care	141	24.6
Primary care hospital	109	19.0
Community pharmacy	45	7.9
Hospital pharmacy	35	6.1
Private hospital	34	5.9
**Working experience**		10.6 ± 9.3 (Median 7, IQR: 18–2)
<5 years	198	34.6
5–10 years	140	24.4
>10 years	235	41.0

*The data are presented as frequency with proportion. The continuous data are showed with mean ± standard deviation along with median (interquartile range)*.

### Prescribing Pattern of FQs

The prescribing pattern of FQs was only evaluated among prescribers (*n* = 262/573, 45.8%). Ciprofloxacin (64.5%), levofloxacin (39.3%), ofloxacin (27.5%), and moxifloxacin (24.8%) were most prescribed FQs by the physicians and dentists. More than half of the prescribers reported that they do not prescribe gemifloxacin, delafloxacin, norfloxacin, gatifloxacin, nalidixic acid, and cinoxacin. Alarmingly, a large number of prescribers reported that trovafloxacin (42.7%), sparfloxacin (37%), cinoxacin (33.6%), lomefloxacin (30.5%), gatifloxacin (26%), nalidixic acid (25.6%), and delafloxacin (23.7%) are new drugs, and they were not aware of them before administering this survey (Table 2).

**Table 2:** Prescribing pattern of fluoroquinolones among prescribers (physicians and dentists), *N* = 262.

**Drugs**	**I do not prescribe this drug**		**I prescribe this drug frequently**		**I prescribe this drug less frequently**		**This drug is new for me**	
	**Frequency**	**Percentage**	**Frequency**	**Percentage**	**Frequency**	**Percentage**	**Frequency**	**Percentage**
Ciprofloxacin	25	9.5	169	64.5	68	26.0	0	0.0
Levofloxacin	52	19.8	103	39.3	107	40.8	0	0.0
Ofloxacin	76	29.0	72	27.5	114	43.5	0	0.0
Moxifloxacin	96	36.6	65	24.8	85	32.4	16	6.1
Gemifloxacin	163	62.2	2	0.8	58	22.1	39	14.9
Delafloxacin	138	52.7	7	2.7	55	21.0	62	23.7
Norfloxacin	146	55.7	15	5.7	62	23.7	39	14.9
Gatifloxacin	139	53.1	9	3.4	46	17.6	68	26.0
Lomefloxacin	127	48.5	11	4.2	44	16.8	80	30.5
Nalidixic.acid	147	56.1	5	1.9	43	16.4	67	25.6
Cinoxacin	134	51.1	4	1.5	36	13.7	88	33.6
Travofloxacin	114	43.5	2	0.8	34	13.0	112	42.7
Sparfloxacin	120	45.8	10	3.8	35	13.4	97	37.0

### Participant's Knowledge Regarding Indications and AEs of FQs

The average knowledge score for indications was 5.29 ± 3.05 (Table 3). The highest score was achieved by the physicians followed by pharmacists. Inter-professional comparisons showed that physicians and pharmacists scored significantly higher than nurses (*p* < 0.001 and *p* = 0.006, respectively), but inferences were insignificant when their scores were compared with dentists (Table 4). The most commonly reported indications were UTIs (77%) followed by bacterial eye infection (63.4%), typhoid fever (54.1%), respiratory tract infections (RTIs) (50.3%), gonorrhea (49%), otitis externa (44.3%), and skin and soft tissue infections (SSTIs) (42.2%). About one-fourth of the participants reported that FQs are not indicated for surgical prophylaxis, meningococcal meningitis, and SSTIs. More than half of the study population were not sure that FQs are indicated for anthrax and fistulating Crohn's disease (Table 3). Although FQs are prescribed for all listed twelve indications, none of the respondents selected all from the list.

**Table 3:** Participants' knowledge toward indications and adverse effects of fluoroquinolones.

	**Yes**		**No**		**Not sure**		**Item score**
	**Frequency**	**Percentage**	**Frequency**	**Percentage**	**Frequency**	**Percentage**	**Score (SD)**
**Indications**
Bacterial eye infection	363	63.4	54	9.4	156	27.2	0.63 ± 0.48
Corneal ulcer	180	31.4	127	22.2	266	46.4	0.31 ± 0.46
Otitis externa	254	44.3	102	17.8	217	37.9	0.44 ± 0.50
Respiratory tract infections (RTIs)	288	50.3	44	7.7	241	42.1	0.50 ± 0.50
Urinary tract infections	441	77.0	53	9.2	79	13.8	0.77 ± 0.42
Skin & soft tissue infections	242	42.2	144	25.1	187	32.6	0.42 ± 0.49
Gonorrhea	281	49.0	92	16.1	200	34.9	0.49 ± 0.50
Surgical prophylaxis	171	29.8	156	27.2	246	42.9	0.30 ± 0.46
Anthrax	179	31.2	88	15.4	306	53.4	0.31 ± 0.46
Meningococcal meningitis	174	30.4	149	26.0	250	43.6	0.30 ± 0.46
Fistulating Crohn‘s Disease	157	27.4	111	19.4	305	53.2	0.27 ± 0.45
Typhoid fever	310	54.1	71	12.4	192	33.5	0.54 ± 0.50
Average knowledge score for indications domain (out of 12)							**5.29** **±3.05**
**Adverse effects**
Nauseas or vomiting	460	80.3	19	3.3	94	16.4	0.80 ± 0.40
Diarrhea	412	71.9	43	7.5	118	20.6	0.72 ± 0.45
Arthropathy	214	37.3	91	15.9	268	46.8	0.37 ± 0.48
Loss of appetite	113	19.7	122	21.3	338	59.0	0.20 ± 0.40
Musculoskeletal pain	250	43.6	61	10.6	262	45.7	0.44 ± 0.50
Renal impairment	213	37.2	105	18.3	255	44.5	0.37 ± 0.48
Hepatic impairment	116	20.2	72	12.6	385	67.2	0.20 ± 0.40
Headache	267	46.6	94	16.4	212	37.0	0.47 ± 0.50
Dizziness	254	44.3	98	17.1	221	38.6	0.44 ± 0.50
Seizures	122	21.3	125	21.8	326	56.9	0.21 ± 0.41
Dyspnea	127	22.2	126	22.0	320	55.8	0.22 ± 0.42
Trouble sleeping	99	17.3	122	21.3	352	61.4	0.17 ± 0.38
Altered smell sensation	109	19.0	132	23.0	332	57.9	0.19 ± 0.39
Asthenia	53	9.2	146	25.5	374	65.3	0.09 ± 0.29
Sensation abnormalities (peripheral neuropathy)	119	20.8	120	20.9	334	58.3	0.21 ± 0.41
Clostridium difficile infections	156	27.2	114	19.9	303	52.9	0.27 ± 0.45
Cardiovascular problems	177	30.9	109	19.0	287	50.1	0.31 ± 0.46
Photosensitivity or skin reactions	274	47.8	75	13.1	224	39.1	0.48 ± 0.50
Average knowledge score for adverse effects domain (out of 18).							6.17 ± 4.05

**Table 4:** Comparison of knowledge score toward indications and adverse effects across different specialties of healthcare professionals.

**Specialty of HCPs**	**Knowledge score**	***P*-Value (vs. physicians)**	***P*-Value (vs. pharmacists)**	***P*-Value (vs. dentists)**	***P*-Value (vs. nurses)**
**Indication score**
Physicians	5.70 ± 2.77	–	0.759	0.208	**<0.001**
Pharmacists	5.42 ± 2.71	–	–	0.465	**0.006**
Dentists	4.59 ± 3.24	–	–	–	0.918
Nurses	4.20 ± 4.01	–	–	–	–
**Adverse effects score**
Physicians	6.38 ± 3.85	–	0.940	0.064	0.090
Pharmacists	6.59 ± 3.33	–	–	**0.031**	**0.030**
Dentists	4.50 ± 4.32	–	–	–	0.820
Nurses	5.22 ± 5.54	–	–	–	–

*The bold values indicate significant results and their values is less than 0.05*.

The average knowledge score for AEs was 6.17 ± 4.05 (Table 3). The highest score was achieved by the pharmacists followed by physicians. Inter-professional comparisons showed that pharmacists scored significantly higher than dentists (*p* = 0.031) and nurses (*p* = 0.030), but inferences were insignificant when their scores were compared with physicians. The most commonly reported AEs were nausea or vomiting (80.3%), diarrhea (71.9%), photosensitivity or skin reactions (47.8%), headache (46.6%), dizziness (44.3%), musculoskeletal pain (43.6), arthropathy (37.3%), renal impairment (37.2%), and cardiovascular problems (30.9%) (Table 3). More than half of the respondents were not sure that hepatic impairment, asthenia, trouble sleeping, loss of appetite, sensation abnormalities, altered smell sensation, seizures, dyspnea, *Clostridium difficile* infections, and cardiovascular problems are the AEs of FQs. Although the use of FQs is associated with all of these AEs, none of the respondents selected them all from the list. Alarmingly, 217 (37.9%) respondents did not identify the correct mechanism of action of FQs (average score: 0.62 ± 0.49), where most of the respondents were dentists (81.2%) and nurses (89.1%).

### Participants' Awareness and Knowledge Toward BWs of FQs

**Figure 2:**
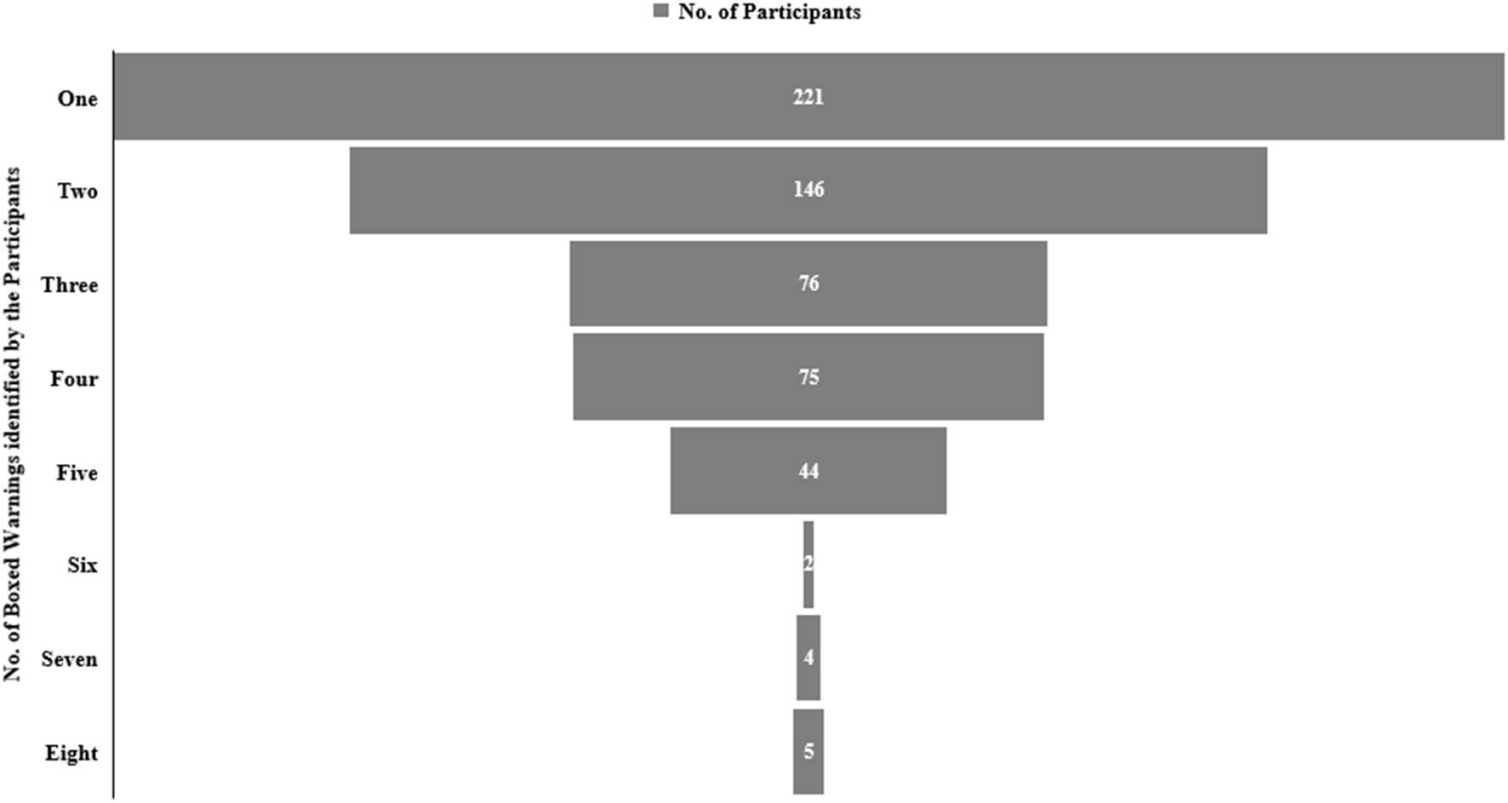
The frequency distribution on the selection of boxed warnings (BWs) against FQs among study participants.

The majority of the participants (*n*: 356, 62.1%) were not aware that the most recent BW on FQs was issued in 2018. The participants were asked to identify the BW from the list of eight warnings issued by the FDA. The aortic aneurysm was recognized by most of the respondents (45.9%) followed by tendinitis or tendon rupture (38.7%) and hypoglycemia (36.5%) (Table 5). Alarmingly, only 5 participants (0.9%) identified all eight BW, while 4 (0.7%) and 2 (0.3%) participants recognized 7 and 6 warnings, respectively. Only 75 (13.1%) respondents recognized half of the BW from the list. The proportion of respondents who identified at least one warning was 38.6% (Figure 2). The average knowledge score for BW was 2.3 ± 1.5. The knowledge score for BW among physicians significantly differed (*p* = 0.028) from pharmacists and nurses, but not from dentists (*p* = 0.896). These findings showed that prescribers had a higher knowledge score for BW than non-prescribers.

**Table 5:** Participant's knowledge toward boxed warnings on fluoroquinolones.

**FDA boxed warnings on fluoroquinolones**	**Yes**		**No**		**Not sure**	
	***N***	**%**	***N***	**%**	***N***	**%**
Worsening of pre-existing myasthenia gravis	111	19.4	272	47.5	190	33.2
Disabling side effects of the tendons, muscles, nerves and joints	167	29.1	238	41.5	168	29.3
Hypoglycemia	209	36.5	215	37.5	149	26.0
Tendinitis or tendon rupturing	222	38.7	144	25.1	207	36.1
Restricted use of fluoroquinolones for certain uncomplicated infections	92	16.1	275	48.0	206	36.0
Aortic aneurysm/raptures or tears in aorta	263	45.9	205	35.8	105	18.3
Mental health side effects	171	29.8	213	37.2	189	33.0
Peripheral neuropathy	106	18.5	230	40.1	237	41.4
Average knowledge score for BW domain (out of 8)	2.3 ± 1.5					

### Association of Demographics With Knowledge Score

The total knowledge score among HCPs was 14.8 ± 6.4, where only 21.5% had appropriate knowledge (score ≥ 50%). Of these, most of the respondents were working in public sectors (*n* = 110, *p* = 0.003) and had working experience of >10 years (*n* = 63, <0.001). The score was significantly higher among HCPs with age >40 years (*p* = 0.039), Indian nationals (*p* = 0.001), expatriates (*p* < 0.001), HCPs working in Riyadh and Eastern regions, physicians and pharmacists (*p* < 0.001), and those who were practicing in public sectors (*p* = 0.003). In addition, the HCPs having working experience of <5 years had significantly (*p* < 0.001) lower knowledge scores than those with working experience of >5 years. However, knowledge scores did not differ significantly among the gender and education levels of the participants. Table 6 indicates the average knowledge score of demographic parameters along with Tukey's honestly significant difference (HSD) *post-hoc* analysis. The adjusted logistic regression analysis found that the HCPs working in Riyadh (OR: 7.7) and Eastern region (OR: 5.8), and those with working experience of 5–10 years (OR: 2.7) and >10 years (OR: 2.4) were associated with the propensity of achieving 50% or above knowledge score (Table 7).

**Table 6:** Predictors of achieving ≥50% knowledge score.

**Variables**	**Univariate**		**Multivariate**	
	**OR (95% Cl)**	***P*-Value**	**OR (95% Cl)**	***P*-Value**
**Age**
20–40 years	1.00 (Reference)			
>40 years	0.803 (0.518–1.245)	0.328		
**Gender**
Male	1.00 (Reference)			
Female	1.490 (0.997–2.226)	0.052		
**Nationality**
Saudis	1.00 (Reference)			
Non-Saudis (expatriates)	1.356 (0.900–2.043)	0.145		
**Provinces**
Al-Jouf region	1.00 (Reference)			
Riyadh region	6.603 (3.502–10.497)	**<0.001**	7.717 (4.388–13.575)	**<** **0.001**
Eastern region	7.623 (4.398–13.214)	**<0.001**	5.786 (3.309–10.116)	**<0.001**
Northern Border region	0.535 (0.181–1.581)	**0.258**	0.536 (0.180–1.595)	**0.262**
**Field of education**
Medicine (MBBS/MD)	1.00 (Reference)			
Pharmacy	1.181 (0.757–1.842)	0.465		
Dentistry	0.392 (0.115–1.343)	0.136		
Nursing	1.053 (0.585–1.898)	0.863		
**Level of education**
Graduate	1.00 (Reference)			
Postgraduate	0.659 (0.431–1.007)	0.054		
**Country of graduation**
Saudi Arabia	1.00 (Reference)			
Other countries	1.105 (0.738–1.654)	0.629		
**Working sector**
Government sector	1.00 (Reference)			
Private sector	0.408 (0.221–0.756)	**0.004**	0.599 (0.297–1.208)	0.152
**Working experience**
<5 years	1.00 (Reference)		1.00 (Reference)	
5–10 years	2.980 (1.670–5.318)	**<0.001**	2.701 (1.426–5.117)	**0.002**
>10 years	2.930 (1.727–4.973)	**<0.001**	2.430 (1.324–4.4.460)	**0.004**

*The bold values indicate significant results and their values is less than 0.05*.

**Table 7:** Association of participant's demographics with knowledge score.

	**Mean SD**	**Percentage (%)**
**Age**		0.039
20 – 40 Years	14.4 ± 6.7	
>40 Years	15.6 ± 5.9	
**Gender**		0.523
Male	14.7 ± 6.1	
Female	15.0 ± 6.9	
**Nationality**		0.001
Saudis	14.1 ± 6.8	
Egyptians	15.6 ± 5.2	
Sudanese	14.9 ± 3.5	
Pakistanis	15.0 ± 5.3	
Indians	18.5 ± 7.1	
Syrians	17.1 ± 3.4	
**Saudis vs. Non-Saudis**		<0.001
Saudis	14.1 ± 6.8	
Non-Saudis (expatriates)	16.1 ± 5.5	
**Provinces**		<0.001
Al-Jouf region	13.8 ± 5.6	
Riyadh region	17.9 ± 7.4	
Eastern region	17.5 ± 5.2	
Northern Border region	10.2 ± 5.8	
**Field of education**		<0.001
Medicine (MBBS/MD)	15.7 ± 5.6	
Pharmacy	15.4 ± 5.5	
Dentistry	12.6 ± 6.5	
Nursing	12.0 ± 9.0	
**Level of education**		0.781
Graduation	14.7 ± 7.1	
Master	15.1 ± 5.1	
Doctorate	14.9 ± 5.4	
**Country of graduation**		0.001
Saudi Arabia	14.1 ± 7.0	
Egypt	15.8 ± 5.1	
India	18.5 ± 7.1	
Pakistan	15.4 ± 5.4	
Sudan	15.2 ± 3.5	
Syria	17.1 ± 3.4	
UK	12.3 ± 2.6	
USA	14.2 ± 6.1	
**Working sector**		0.003
Government sector	15.2 ± 6.5	
Private sector	13.2 ± 6.1	
**Working organization**		<0.001
Tertiary care hospital	17.0 ± 6.8	
Secondary hospital care	13.4 ± 5.1	
Primary care hospital	14.1 ± 6.5	
Community pharmacy	14.9 ± 4.7	
Hospital pharmacy	11.9 ± 6.4	
Private hospital	12.4 ± 7.0	
**Working experience**		<0.001
<5 years	12.9 ± 6.4	
5–10 years	15.8 ± 5.4	
>10 years	15.9 ± 6.7	

*The data are presented as frequency with proportion. The continuous data are shown with mean ± standard deviation along with median (interquartile range)*.

*Post-hoc analysis (Tukey's HSD): Nationality, Saudis vs. Indians (p = 0.001); Provinces, Al-Jouf regions vs. Riyadh region (p < 0.001); Al-Jouf regions vs. Eastern region (p < 0.001), Al-Jouf regions vs. northern border region (p < 0.001), Field of education, MBBS/MD vs. nursing (p < 0.001); MBBS/MD vs. dentistry (p = 0.045), pharmacy vs. nursing (p < 0.001), Country of Graduation, India vs. KSA (0.001), India vs. UK (0.016); Working Organization, Tertiary care hospital vs. Secondary care hospital (p < 0.001), Tertiary care hospital vs. Primary care hospital (p = 0.001), Tertiary care hospital vs. Hospital pharmacy (p < 0.001), Tertiary care hospital vs. Private hospital (p = 0.001); Working experience, <5 years vs. 5–10 years (p < 0.001), <5 years vs. >10 years (p < 0.001)*.

## Discussion

To the best of our search, this is the first large-scale study to explore the awareness and extent of knowledge about safety profile, usage, and FDA BWs of FQs among HCPs in the KSA. The “BWs.” formerly known as “black box warnings,” are the most serious safety-related warnings instituted by the FDA in 1979. These warnings are intended to inform the HCPs regarding cautious decision-making during the treatment. Alarmingly, more than 600 medications have BW and about 40% of patients receive at least one such drug. The continuous increase in the number of BW warrants HCPs to remain up to date on the safety profile of drugs. However, recent data indicate that a series of BW against FQs did not impact the prescribing pattern of these drugs among HCPs across the world (2, 5, 7, 10–12). The non-adherence to BW among prescribers might be attributed to the lack of knowledge of such warnings. Smollin *et al*. reported that physicians in emergency and pediatric departments had limited knowledge of medications with BW or the content of such warnings. The authors reported that only 36.3% of physicians had correctly identified the drugs with BW from the list of 15 medications (19). The violation of BW has also been observed in another study where 7 of 1,000 patients received a prescription having drugs with BW (13).

The FQs belong to the list of the world's most commonly prescribed antibiotics. In 2014, FQs were marked as the fourth most commonly prescribed class of antibiotics in the United States (20). In KSA, the use of FQs accounted for up to 19% of antimicrobial prescriptions in ambulatory care and outpatient departments (1). The safety of FQs is under investigation for the last two decades amid several reports on their association with disabilities among users (20). In this context, it was imperative to ascertain the knowledge of HCPs regarding the safety profile of these drugs in KSA, specifically when FQs prescription rates are very high in the country.

Alarmingly, HCPs indicated unsatisfactory knowledge in this study, where only one-fifth of participants have achieved ≥50% knowledge score. The results are consistent with the findings of another study where only one-third of physicians were able to identify the drugs with BW (19). These findings necessitate the need for urgent measures from health authorities in terms of educational campaigns and antibiotic stewardship programs (ASP).

About 46% of the participants in this study were prescribers, and ciprofloxacin was reported as the most prescribed drug followed by levofloxacin. Alarmingly, a large proportion of the prescribers were not aware of certain drugs from FQs class, such as trovafloxacin, sparfloxacin, cinoxacin, lomefloxacin, gatifloxacin, nalidixic acid, and delafloxacin. Our analysis showed that prescribers were only familiar with ciprofloxacin, levofloxacin, and ofloxacin. Nalidixic acid, discovered in 1962 and gained its clinical importance in 1967, was the first agent from FQs class (21). However, 25% of prescribers did not recognize this drug as a member of FQs class, indicating the lack of basic concepts which should be considered as a serious concern for the healthcare authorities. These results urge the need for continuous education programs emphasizing the introduction and core concepts of antimicrobials.

Our findings indicate that the knowledge of the HCPs for indications and AEs of FQs was unsatisfactory. The interprofessional comparisons showed that physicians and pharmacists had better knowledge of indications and AEs of FQs as compared to dentists and nurses. The UTIs were reported by most of the participants as indications of FQs, followed by bacterial eye infection and typhoid fever. It is pertinent to mention that a large proportion of the participants reported that surgical prophylaxis, meningitis, and SSTIs are not indications for FQs. A single dose of ciprofloxacin (500 mg) before the procedure is an effective prophylactic regime (22). In addition, the clinical practice guidelines for antimicrobial prophylaxis in surgery also suggest the use of FQs within 120 min of the procedure (23). Similarly, the efficacy of ciprofloxacin has been well-established for the treatment or prophylaxis of meningococcal meningitis (24). However, the ciprofloxacin-resistant strain of *Neisseria meningitidis* has been evolved in recent years (25). Moreover, the FQs have proved as a promising addition to the current armamentarium against SSTIs with an additional benefit of dosage convenience (26). The HCPs were also asked to select the AEs of FQs from the list of eighteen. The most commonly reported AEs were nausea, vomiting, diarrhea, headache, dizziness, skin reactions, and musculoskeletal pain. However, a large proportion of HCPs were not able to identify other AEs. The use of FQs is broadly linked with AEs of all major organ systems, including cardiovascular (18.6%), central nervous (11.9%), peripheral nervous (6.8%), gastrointestinal (15.3%), musculoskeletal (6.8%), and dermatological (20.3%) systems (27, 28). A recent umbrella review investigated the relationship of FQs with emerging AEs through pooling the data from various meta-analyses. The authors ascertained that the use of FQs is markedly associated with neuropsychiatric toxicities and cardiovascular events, including aortic dissection, tendon rupture, and retinal detachment (29). In our study, about 80% of participants were either disagreed or not sure that asthenia, trouble sleeping, altered smell sensation, loss of appetite, hepatic impairment, sensation abnormalities, seizures, and dyspnea are the AEs of the FQs. It is pertinent to mention that all the listed AEs in the study tool are present in product literature or British National Formulary and categorized as common or uncommon side effects (30). Taken together, this study underscored that the HCPs lack sufficient information on the use and safety of the FQs. These findings trigger the need for regular training in hospitals and community pharmacies. In addition, these training campaigns could be considered a prerequisite for licensure renewal to ensure that the HCPs have up-to-date information regarding optimal disease management.

Our results underscored poor participants' knowledge toward the BW, reflected by the finding that 62% of HCPs were not aware that the most recent BW against FQs was issued in 2018. The aortic aneurysm was selected by most of the HCPs (45.9%) as a BW of FQs. Of the list of eight, half of the BWs were selected by only 13% HCPs, while one-third of participants identified at least one warning. Unfortunately, we did not come across any study evaluating the knowledge of BW of FQs among HCPs for direct comparison with our findings. However, few studies have evaluated the extent of awareness and recognition of BW among HCPs, regardless of the drugs. These studies have shown insufficient knowledge of physicians and pharmacists toward the BW (19, 31). Other studies investigating the knowledge of BW among pharmacy and medical students reported a low level of knowledge which was directly proportionate to their study years (32, 33). Since lack of information regarding BW may put patients at increased risks, the FDA along with other health authorities should adopt an appropriate mechanism of communication for such warnings and should also develop systematic feedback to ensure that all BW are communicated to HCPs across the world. Although we did not find any study with similar objectives assessed in this study, other recent investigations have reported unsatisfactory knowledge among HCPs toward the reporting of adverse events and basics of pharmacovigilance (34–37).

The exact mechanisms underlying the serious AEs precipitated by the FQs are under debate. However, FQs-induced mitochondrial toxicities due to oxidative stress across a range of mammalian cells are considered the most plausible pathogenesis for serious AEs (20).

Though this study reported a considerably low level of knowledge among all categories of HCPs, the knowledge score was comparatively higher among physicians and pharmacists than dentists and nurses. Similarly, the HCPs working in public sectors (hospitals) had higher knowledge scores than those working in private sectors (e.g., private hospitals and community pharmacies). In addition, the working experience was positively associated with the knowledge among study participants. Interestingly, the HCPs working in Riyadh and Eastern regions demonstrated better knowledge as compared to those from Al-Jouf and Northern Border regions. The regional disparities in knowledge score might be attributed to the preference of competent professionals to pursue their jobs in Riyadh and Eastern regions, as these two regions are more developed and have the state-of-the-art health facilities. However, the Northern border and Al-Jouf regions are least developed with limited health facilities, and the HCPs from these regions may have fewer opportunities for continuous learning (38). Where this study urges the need for educational campaigns for HCPs, at the same time, our results underscored the targeted groups of HCPs requiring dire attention of the health authorities. These factors must be considered if health authorities decide to initiate targeted or selected educational programs.

Our analysis carries a few limitations which should be considered while interpreting the results. We used a self-report as the main method of inquiry, which may have introduced recall bias. The findings of this study are, however, limited to HCPs from the four provinces of KSA and may not be generalized in a broader context. However, the results can be implicated to other provinces as study participants were included from developed, developing, and underdeveloped provinces of the KSA. The proportion of responses from dentists and nurses was comparatively less which may bias the findings toward clinicians and pharmacists. This shortcoming (selection bias) underscores the consideration of equal response proportion in future investigations. The knowledge evaluation in this study was based on difficult and detailed items, and there is a high propensity that questions or items of a medium-level difficulty may yield better scores among participants. The choice of indications and AEs included in the survey could be less familiar to some of the participants as HCPs may only know the indications or AEs which are specific to certain agents of FQs approved in their health facilities. However, these indications and AEs are explicitly included in the product's leaflets and popular tertiary information sources. Moreover, the HCPs may feel reluctant to answer the questions honestly amid the threats of professional self-esteem, reputation, and perceiving them as incompetent. We aim to mitigate such bias by administering the survey in an anonymous fashion. In addition, we requested the participants to avoid any information source to get the answer in order to ensure the assessment of their inherent knowledge. Nevertheless, this study is strengthened by the first large-scale analysis including diverse categories of the HCPs from various disciplines. The findings of this study will serve to design and implement medication-related knowledge initiatives for HCPs by the ministry of health and Saudi FDA (SFDA).

## Conclusion

This study underscored that HCPs working in KSA have inadequate knowledge toward safety profile, use, and BW of FQs which may put patients at increased risks of adverse events. Interprofessional comparisons showed that physicians and pharmacists have higher knowledge scores than dentists and nurses. In addition, subgroup analysis indicated a significant association of age, nationality, working location or sector, country of graduation, field of education, and working experience with the knowledge score. There is a dire need to initiate antimicrobial-focused educational campaigns for HCPs. Moreover, methods to improve education and disseminate FDA warnings in practice are needed. Our findings also necessitate the need for more comprehensive and elaborative surveys to ascertain the familiarity of HCPs with the safety profiles of the high-risk drugs.

## Data Availability Statement

The original contributions presented in the study are included in the article/supplementary material, further inquiries can be directed to the corresponding author/s.

## Ethics Statement

The studies involving human participants were reviewed and approved by Local Committee of Bioethics, Jouf University. The patients/participants provided their written informed consent to participate in this study.

## Author Contributions

THM, ASAla, YHK, and NHA: conception or design of the work. MS, AIA, S-U-DK, ADA, NKA, and ASAle: analysis or interpretation of data for the work. MS, AIA, ADA, NKA, ASAle, and MSI: data collection. YHK, MS, ZI, S-U-DK, and MHB: drafting the work. THM, ASAle, NHA, ASAla, and MSI: revising the manuscript critically for important intellectual content. All authors provided approval for publication of the content and are accountable for all aspects of the work and contributed to the article and approved the submitted version.

## Funding

The authors extend their appreciation to the Deanship of Scientific Research at Jouf University for funding this work through research Grant No. DSR2020-04-2565.

## Conflict of Interest

The authors declare that the research was conducted in the absence of any commercial or financial relationships that could be construed as a potential conflict of interest.

## Publisher's Note

All claims expressed in this article are solely those of the authors and do not necessarily represent those of their affiliated organizations, or those of the publisher, the editors and the reviewers. Any product that may be evaluated in this article, or claim that may be made by its manufacturer, is not guaranteed or endorsed by the publisher.
